# Risk analysis for patients with arterial thromboembolic events after intravitreal ranibizumab or aflibercept injections

**DOI:** 10.1038/s41598-023-34128-5

**Published:** 2023-05-10

**Authors:** Yun-I. Chou, Hao-Yun Chang, Meng-Yin Lin, Ching-Han Tseng, Tsung-Jen Wang, I-Chan Lin

**Affiliations:** 1 Department of Medical Education, Taipei Veterans General Hospital, Taipei, Taiwan; 2 School of Medicine, College of Medicine, Taipei Medical University, Taipei, Taiwan; 3 Department of Ophthalmology, Shuang Ho Hospital, Taipei Medical University, New Taipei City, Taiwan; 4 Department of Ophthalmology, School of Medicine, College of Medicine, Taipei Medical University, Taipei, Taiwan; 5 Department of Ophthalmology, Taipei Medical University Hospital, Taipei Medical University, Taipei, Taiwan; 6 Department of Ophthalmology, Wan Fang Hospital, Taipei Medical University, Taipei, Taiwan

**Keywords:** Diseases, Health care, Risk factors

## Abstract

Intravitreal anti–vascular endothelial growth factor (anti-VEGF) agents have been increasingly applied in the treatment of retinal neovascular diseases. Concerns have arisen that these intravitreal agents may be associated with a potential risk of arterial thromboembolic (ATE) events. We conducted a retrospective, nationwide population‐based cohort study to analyze the risks for ATE events in patients receiving intravitreal ranibizumab (IVR) or intravitreal aflibercept (IVA). Data (2011–2018) were obtained from Taiwan’s National Health Insurance Research Database. Cox proportional-hazards model was used to identify the risk factors for ATEs. Of the total 3,469 patients, 1393 and 2076 patients received IVR and IVA, respectively. In our result, 38 ATEs occurred within 6 months after IVR or IVA. The risk of ATEs was lower in patients receiving IVR than in those receiving IVA (adjusted hazard ratio [aHR], 0.27; 95% confidence interval [CI], 0.11–0.66). Patients with coronary artery disease (CAD) exhibited a higher risk of ATEs than did those without CAD (aHR, 3.47; 95% CI, 1.41–8.53). The risk of ATEs was higher in patients with an event of acute myocardial infarction (AMI) or ischemic stroke (IS) within 6 months prior to index IVI than in those without recent AMI/IS events (aHR, 23.8; 95% CI, 7.35–77.2 and IS: aHR, 290.2; 95% CI, 103.1–816.4). In conclusion, compared with IVA, IVR was associated with a lower risk of ATEs. When strategies for anti-VEGF agents are devised, risk factors, such as CAD and a history of AMI or IS within 6 months should be considered. Further large-scale studies are warranted to elucidate the safety of anti-VEGF injections.

## Introduction

Vascular endothelial growth factor (VEGF) is considered to be the primary regulator of ocular neovascularization due to retinal hypoxia^1^. Neovascular age-related macular degeneration (nAMD), retinal vein occlusion (RVO), and diabetic macular edema (DME) are the most prominent angiogenesis-driven ocular diseases that cause vision loss and blindness, thus posing a significant economic burden on health care^2^. Fortunately, the advent of anti-VEGF agents has ushered in a pharmacotherapeutic revolution with regard to the aforementioned retinal neovascular diseases^3^.

Administration of several intravitreal anti-VEGF agents has become routine clinical practice. The three most commonly used anti-VEGF drugs are ranibizumab (Lucentis^®^, Genentech Inc., South San Francisco, CA, USA), aflibercept (Eylea^®^, Regeneron Pharmaceuticals Inc., Tarrytown, NY, USA), and bevacizumab (Avastin^®^, Genentech Inc., South San Francisco, CA, USA). Ranibizumab, a recombinant humanized monoclonal antibody against all isoforms of VEGF-A, was first approved by the US Food and Drug Administration (FDA) in 2006 and by the Taiwan FDA in 2009. Aflibercept, a recombinant fusion protein that binds with high affinity not only to VEGF-A, but also to VEGF-B and placental growth factor (PlGF), was approved by the US FDA in 2011, and Taiwan FDA in 2013^4^.

VEGF stimulates the production of nitric oxide, which exerts several vasculoprotective effects, such as vasodilation, antithromboticity, and angiogenesis. Therefore, systemic administration of anti-VEGF agents is associated with arterial thromboembolic events (ATEs) and hypertension^5–8^. Several studies have indicated increased risks of adverse cardiovascular events with the IVI (intravitreal injection) of anti-VEGF agents, particularly in patients with risk factors such as old age, diabetes, and an ATE history^9–13^. In addition, patients with an acute myocardial infarction (AMI)/ischemic stroke (IS) history, particularly within 1 year before the IVI of anti-VEGF agents, exhibit higher rates of mortality than those without AMI/IS events before IVI^14^. Therefore, safety measures must be adopted for high-risk patients receiving IVI of anti-VEGF agents. In the present retrospective, nationwide population cohort study, we analyzed real-world data to identify the risk factors for ATEs in patients receiving IVI of anti-VEGF agents.

## Methods

### Data source

We obtained data from the Longitudinal Generation Tracking Database (LGTD 2005), which is maintained by the Health and Welfare Data Science Center (HWDC) in Taiwan. The LGTD 2005 that includes 2 million people who were randomly sampled from all beneficiaries in Taiwan's National Health Insurance (NHI) program by the HWDC. The NHI program is a mandatory health insurance program that covered more than 99% of all legal residents of Taiwan^15^.The HWDC has verified no significant difference in age, gender, region, ambulatory care, and inpatient expenditures between the LGTD2005 and the compulsory universal NHI program^15^.

Diseases were defined using the *International Classification of Diseases* (ICD), *Ninth Revision*, *Clinical Modification* (ICD-9-CM) and ICD, *Tenth Revision*, *Clinical Modification* (ICD-10-CM). The data are available exclusively for research; confidentiality must be maintained according to the directives of the National Health Insurance administration. This study protocol was approved by the Taipei Medical University–Joint Institutional Review Board (TMU-JIRB N202104116) and the Data Release Review Board of the Health Promotion Administration, Ministry of Health, and Welfare in Taiwan. All methods were performed in accordance with the relevant guidelines and regulations. In addition, the Taipei Medical University–Joint Institutional Review Board waived the requirement for informed consent due to the lack of personal information and secondary data in the study.

### Study population

**Figure 1:**
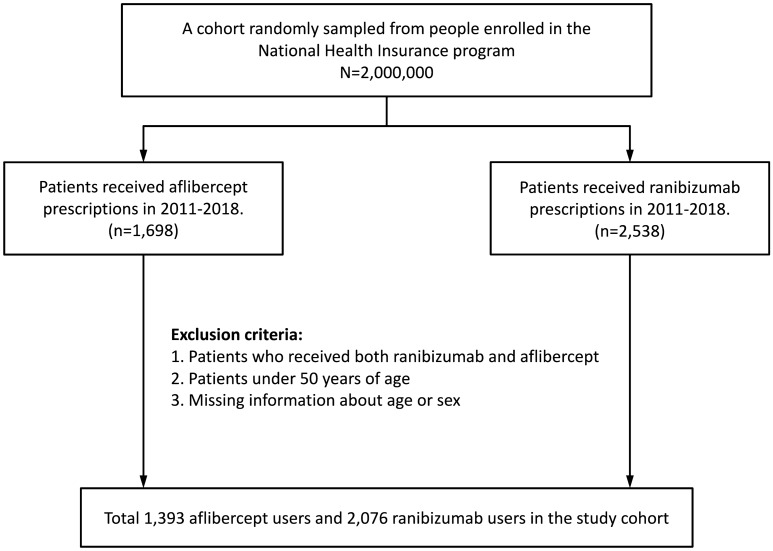
Patients’ selection process.

The present study included patients aged > 50 years who received either intravitreal ranibizumab (IVR) (ranibizumab: Anatomical Therapeutic Chemical [ATC] code S01LA04) or aflibercept (IVA) (aflibercept: ATC code S01LA05) injections between 2011 and 2018. People who received both IVR and IVA during the aforementioned period were excluded. The index date was the date of the first prescription of ranibizumab or aflibercept. The follow-up period was 2011–2018. Study outcome data until December 31, 2018, were collected to ensure that at least 6-month follow-up data were available for all eligible patients. This study included patients with at least 1-year lookback data before the index injection and without any history of anti-VEGF agents’ injection during the previous year. Patients were censored before 180 days after the index IVI of ranibizumab or aflibercept if they received any other anti-VEGF agents or died. Figure 1 showed the flowchart of patient selection process.

### Primary outcome and covariates

The primary study outcome was the occurrences of ATEs, which included IS; ICD-9-CM codes 433–436; ICD-10-CM codes I63, I65, I66, and I6789) and acute myocardial infarction (AMI; ICD-9-CM code 410; ICD-10-CM codes I21 and I22).IS and AMI events that occurred within 6 months following intravitreal anti-VEGF agents were included. We adjusted for likely covariates, such as hypertension (ICD-9-CM codes 401–405; ICD-10-CM codes I10, I11, I12, I13, I15, and N262), diabetes (ICD-9-CM code 250; ICD-10-CM codes E08, E09, E10, E11, and E13), renal disease (ICD-9-CM codes 585, 586, 403, and 404; ICD-10-CM codes I120, I129, I13, N184, N185, N186, N189, and N19), atrial fibrillation (ICD-9-CM code 427.3; ICD-10-CM code I48), and coronary artery disease (CAD; ICD-9-CM codes 413, 414, and 429.2; ICD-10-CM codes I201, I251, I253, I254, I255, I256, I257, I25810, I25811, I25812, I2589, and I259). Comorbidities occurring within 12 months before the index IVR or IVA were defined using primary or secondary ICD-9 or ICD-10 codes. We specifically recorded the AMI and IS events occurring within 6 months before the index IVI. AMI events were diagnosed according to the ICD-9-CM or ICD-10-CM diagnostic codes, hospitalization, and antiplatelet therapy administration. IS events were diagnosed according to the ICD-9-CM or ICD-10-CM diagnostic codes, hospitalization and brain imaging (either computed tomography or magnetic resonance imaging).

IVR and IVA were indicated for nAMD (ICD-9-CM code 362.52; ICD-10-CM code H35.32x), DME (ICD-9-CM code 362.01–07 and 250.x; ICD-10-CM codes E0.83x, E10.3x, E11.3x, and E13.3x), RVO (ICD-9-CM code 362.35–36; ICD-10-CM codes H34.81 × and H34.83x), and myopic choroidal neovascularization (CNV; ICD-9-CM code 362.16; ICD-10-CM code H44.2A). The LGTD 2005 is a reimbursement claims database that covers the residents Taiwan who are enrolled in the NHI program. The NHI program is a single-payer health insurance system that has a contract with most health-care providers and covers 99% of the residents. NHI approval for IVR and IVA reimbursement is based on medical coding, and also includes a review of the applicant’s medical records by retinal specialists. All care providers must upload medical records of reimbursement applications for IVR and IVA.

### Statistical analysis

The rate of incidence was calculated in 1000 person-years. Cox proportional-hazards models were used to calculate hazard ratios (HRs) with 95% confidence intervals (CIs). Adjusted HRs (aHRs) were calculated using multivariable models including age, sex, all comorbidities, presence of ocular diseases and number of IVI. All statistical analyses were performed using SAS (version 9.4), and the results were plotted using R (version 4.0). Statistical significance was set at *p* < 0.05.

## Results

This study included a total of 3469 patients who received IVR or IVA between 2011 and 2018. Among these patients, 1393 (40.10%) and 2076 (59.84%) received ranibizumab and aflibercept, respectively. Table 1 summarizes the demographic and clinical characteristics of patients receiving IVI. Regarding the occurrence ATEs after IVI, 8 (0.23%) AMI events and 30 (0.86%) IS events during the follow-up period. The most common comorbidities were hypertension (75.24%) and diabetes (58.86%). Anti-VEGF injections were indicated mostly for nAMD (44.51%) and DME (34.28%).

**Table 1:** Baseline characteristics of the patients who received anti-VEGF injections between 2011 and 2018.

Intravitreal anti-VEGF agents injection between 2011–2018		
Characteristics	n	%
Age (years)		
50 − 64	1296	37.36
65–74	1130	32.57
≥ 75	1043	30.07
Sex		
Women	1480	42.66
Men	1989	57.34
Anti-VEGF agents		
Ranibizumab	1393	40.16
Aflibercept	2076	59.84
Number of injections		
1	319	9.19
2	465	13.40
3	1338	38.57
≥ 4	1347	38.83
Comorbidities		
Hypertension	2610	75.24
Diabetes	2042	58.86
Renal disease	610	17.58
Atrial fibrillation	124	3.57
CAD	1351	38.94
ATEs within 6 months prior to the index anti-VEGF injections		
AMI	44	1.27
IS	107	3.08
Ocular dieases		
nAMD	1544	44.51
DME	1189	34.28
RVO	399	11.50
Myopic CNV	337	10.87
ATEs occurrence within 6 months after anti-VEGF injections^#^		
AMI	8	0.23
IS	30	0.86

*VEGF* vascular endothelial growth factor; *IVI* intravitreal injection; *CAD* coronary artery disease; *nAMD* neovascular age-related macular degeneration; *DME* diabetic macular edema; *RVO* retinal vein occlusion; *CNV* choroidal neovascularization; *ATEs* arterial thromboembolic events; *AMI* acute myocardial infarction; *IS* ischemic stroke.

^#^6 patients had AMI events, 28 patients had IS events, and 2 patients had both AMI and IS events.

Table 2 presents the results of risk analyses for ATEs after IVI. After adjusting for all covariates, the risk of ATEs after IVI was lower in patients receiving IVR than in those receiving IVA. Patients with CAD or a history of AMI or IS within 6 months before IVI exhibited higher risks of ATEs than did patients without these diseases.

**Table 2:** Risks of occurrences of ATEs after intravitreal anti-VEGF injections.

Variables	Events	PY	Rate^#^	Crude HR (95% CI)	Adjusted HR^†^ (95% CI)
Age (years)					
50 − 64	11	1121	9.81	1.00	1.00
65–74	15	970	14.4	1.47 (0.67–3.25)	0.65 (0.24–1.75)
≥ 75	10	909	12.1	1.23 (0.54–2.84)	0.28 (0.07–1.05)
Sex					
Women	17	1278	13.3	1.00	1.00
Men	19	1721	11.0	0.83 (0.43–1.60)	0.55 (0.24–1.26)
Anti-VEGF agents					
Aflibercept	11	1097	10.0	1.00	1.00
Ranibizumab	25	1903	13.1	1.28 (0.63–2.61)	0.27 (0.11–0.66)**
Comorbidities					
Hypertension					
No	4	730	5.48	1.00	1.00
Yes	32	2270	14.1	2.56 (0.91–7.25)	0.81 (0.19–3.38)
Diabetes mellitus					
No	9	1206	7.46	1.00	1.00
Yes	27	1793	15.1	2.01 (0.94–4.27)	1.64 (0.48–5.64)
Renal disease					
No	24	2477	9.69	1.00	1.00
Yes	12	523	23.0	2.37 (1.19–4.75)*	1.73 (0.78–3.82)
Atrial fibrillation					
No	32	2894	11.1	1.00	1.00
Yes	4	105	37.9	3.45 (1.22–9.74)*	0.73 (0.22–2.42)
CAD					
No	12	1828	6.56	1.00	1.00
Yes	24	1171	20.5	3.12 (1.56–6.24)**	3.47 (1.41–8.53)**
ATEs within 6 months prior to the index ant-VEGF agents injections					
AMI					
No	27	2960	9.12	1.00	1.00
Yes	9	40	226.8	24.6 (11.6–52.3)***	23.8 (7.35–77.2)***
IS					
No	6	2906	2.06	1.00	1.00
Yes	30	93	322.2	125.6 (51.5–306.3)***	290.2 (103.1–816.4)***

*ATEs* arterial thromboembolic events; *VEGF* vascular endothelial growth factor; *CAD* coronary artery disease; *IVI* intravitreal injection; *AMI* acute myocardial infarction; *IS* ischemic stroke; *CI* confidence interval; *HR* hazard ratio; and *PY* person-years.

^#^Incidence rate per 1000 person-years.

^†^Multivariable analysis including age, sex, comorbidities, diagnosed ocular diseases, number of injections, and types of anti-VEGF agent used.

*p < 0.05, **p < 0.01, and ***p < 0.001.

Table 3 presents the incidence rates of ATEs in patients with nAMD, DME, RVO, or myopic CNV, which were 11.2, 16.5, 17.5, or 6.08 per 1000 person-years, respectively. Among them, patients with RVO (aHR, 3.49; 95% CI, 1.01–12.1) exhibited higher risks of ATEs than did those with nAMD (aHR, 1.05; 95% CI, 0.40–2.78), DME (aHR, 1.02; 95% CI, 0.26–4.02), or myopic CNV (aHR, 0.5; 95% CI, 0.11–2.92).

**Table 3:** Risks of occurrences of ATEs in patients receiving intravitreal anti-VEGF injections (stratified by the diagnosed ocular diseases).

Ocular diseases	Events	PY	Rate^#^	Crude HR (95% CI)	Adjusted HR^†^ (95% CI)
nAMD	15	1344	11.2	0.88 (0.45–1.70)	1.05 (0.40–2.78)
DME	22	1330	16.5	1.96 (1.002–3.83)*	1.02 (0.26–4.02)
RVO	5	286	17.5	1.60 (0.62–4.12)	3.49 (1.01–12.1)*
Myopic CNV	4	658	6.08	0.44 (0.16–1.25)	0.57 (0.11–2.92)

*ATEs* arterial thromboembolic events; *nAMD* neovascular age-related macular degeneration; *DME* diabetic macular edema; *RVO* retinal vein occlusion; *CNV* choroidal neovascularization; *CI* confidence interval; *HR* hazard ratio; and *PY* person-years.

^#^Incidence rate per 1000 person-years.

^†^Multivariable analysis including age, sex, comorbidities, diagnosed ocular diseases, number of injections, and types of anti–VEGF agent used.

*p < 0.05, **p < 0.01, and ***p < 0.001.

Regarding the risk of ATEs after intravitreal anti-VEGF agents stratified by the number of injections (Table 4), a high number of IVIs was not associated with a statistically significant increase in the risk of ATEs.

**Table 4:** Risks of occurrences of ATEs in patients receiving intravitreal anti-VEGF injections (stratified by the number of injections).

Number of anti-VEGF injections	Events	PY	Rate^#^	Crude HR (95% CI)	Adjusted HR^†^ (95% CI)
1	3	189	15.9	1.00	1.00
2	8	339	23.6	1.47 (0.39–5.54)	1.47 (0.32–6.77)
3	17	1202	14.1	0.86 (0.25–2.93)	0.76 (0.19–3.08)
≥ 4	8	1269	6.30	0.38 (0.10–1.44)	0.51 (0.11–2.31)

*ATEs* arterial thromboembolic events; *VEGF* vascular endothelial growth factor; *CI* confidence interval; *HR* hazard ratio; and *PY* person-years.

^#^Incidence rate per 1000 person-years.

^†^Multivariable analysis including age, sex, comorbidities, diagnosed ocular diseases, number of injections, and types of anti-VEGF agents used.

*p < 0.05, **p < 0.01, and ***p < 0.001.

## Discussion

In the current study, we investigated the risk factors of ATEs associated with IVI of anti-VEGF agents. We identified that the risk of ATEs was lower in patients receiving IVR than in those receiving IVA. In terms of retinal diseases, this risk was higher in patients with RVO than in those with nAMD, DME, or myopic CNV. In terms of systemic illness, the risk of ATEs was higher in patients with CAD or an AMI or IS event within 6 months before IVA or IVR than in those without these comorbidities.

In the present study, patients receiving IVA exhibited higher risks of ATEs than those receiving IVR. Studies have indicated decreased plasma levels of VEGF after ranibizumab and aflibercept injections, which raises consequently raises the risk of ATEs^16,17^. Ranibizumab is a 48-kDa Fab fragment of a monoclonal antibody against the isoforms of VEGF-A, whereas aflibercept is a 115-kDa Fc fusion protein that binds to not only VEGF-A but also VEGF-B and PlGF^4^. The intact Fc region of aflibercept may extend its half-life, thus leading to decreased serum and plasma levels of VEGF. Aflibercept exhibited the greater reduction in free-VEGF levels than ranibizumab did ^16^. The longer half-life of aflibercept and the higher reduction of serum VEGF levels may be correlated with higher risks of major ATEs in patients receiving aflibercept than in those receiving ranibizumab.

We previously identified hypertension and hyperlipidemia to be the risk factors for RVO ^18^. Patients with RVO are at risks of stroke and cardiovascular diseases^19–22^. Therefore, these patients may have higher risks of ATEs than do those with other ocular diseases, such as nAMD and mCNV. Chang et al.^23^ demonstrated that the incidence of ATEs was higher in patients with DME or RVO than in those with nAMD. Another study revealed that the incidence of systemic adverse events after the anti-VEGF agents injection was higher in patients with RVO than in those with DME or nAMD^24^. The increased risks of ATEs in patients with RVO may be associated with the severity of hypertension and hyperlipidemia. However, the claims database does not include the aforementioned information. Further studies are needed to evaluate the correlation between the risk of ATEs and the severity of hypertension and hyperlipidemia in patients with RVO.

CAD is associated with ischemic heart disease, which may lead to AMI. Moreover, CAD is considered to be a risk factor for cardiotoxicity when patients receive systematic VEGF inhibitors, such as bevacizumab and sunitinib^25^.The intravitreal anti-VEGF agents can access the bloodstream and thus reach detectable levels in the systemic circulation and lead to cardiovascular adverse events^26^. An in vitro study suggested that both ranibizumab and aflibercept markedly increase the levels of atherosclerosis-associated inflammatory mediators in coronary artery endothelial cells, and the proinflammatory effects of aflibercept are even stronger than those of ranibizumab^27^. Inflammatory mediators, such as chemokines and cell adhesion molecules, play crucial roles in atherosclerosis progression^28^. Thus, caution must be exercised before administering these anti-VEGF agents to patients with CAD.

In the present study, patients with AMI/IS events that happened within 6 months prior to anti-VEGF injections exhibited higher risks of ATEs than those without AMI/IS events. Chen et al. indicated higher rates of mortality in patients with a history of AMI/IS receiving intravitreal anti-VEGF agents, particularly those with those events occurred within 1 year prior to IVI, than in patients not receiving these agents^14^. VEGF plays a vital role in mediating angiogenesis, which leads to the reperfusion of ischemic brain tissues after acute stroke^29,30^. Under hypoxic conditions, VEGF mRNA and protein are upregulated^31,32^. In patients with IS, VEGF levels increase markedly after acute stroke on day 1 and peak on days 3 and 7; after 14 days, the serum levels of VEGF are still higher than those individuals having no evidence of stroke^33,34^. Sobrino et al.^35^ reported that elevated serum levels of VEGF could be identified even 3 months after IS attack. Furthermore, the serum levels of VEGF are higher in patients with AMI than in healthy individuals, even during the stable phase after an AMI event^36,37^. Anti-VEGF agent injections decreased systemic VEGF levels^15,38^. The elevated serum levels of VEGF in patients with recent AMI/IS events and suppressive effects of anti-VEGF agents on the serum levels of VEGF in these patients may be associated with the increased risks of ATEs^39^. Further studies are warranted to evaluate the correlation between the changes in the serum levels of VEGF with anti-VEGF agents and the risk of major ATEs; such information may help us determine the “safety period” for high-risk patients receiving anti-VEGF agents.

In the present study, the number of injections was not correlated with the risk of ATEs after IVI. The correlation between the frequency of anti-VEGF injections and the incidence of death was investigated in a meta-analysis, which consists of one study with 7.1 injections within 12 months and another study with 11.6 injections within 24 months. As a result, more frequent anti-VEGF injections do not substantially increase the risk of death, except in patients with DME^40^. Ziemssen et al. indicated that adverse events after ranibizumab injections occurred mostly after 3 or 4 injections^24^. However, 77.78% of patients included in the present study received < 4 injections. The risk may be increased if the injection number more than 4 injections. The reduction of serum VEGF significantly during the first month after receiving IVI of anti-VEGF agents^16^. The higher reduction of serum VEGF levels may be related to the more frequent injections. Further studies are needed to assess the risk of ATEs in patients receiving anti-VEGF agents at high doses and frequencies, particularly those at a high risk of major ATEs.

The strength of our study are as follows. We used real-world data and specifically analyzed some risk factors for major ATEs after IVR or IVA. Patients with chronic retinal diseases require multiple injections over a certain period, and the risk of major ATEs must be considered in patients with systemic diseases. Although some studies demonstrated that intravitreal anti-VEGF agents did not increase the risks of ATEs comparing to the non-IVI groups, patients with cardiovascular diseases, or patients with an AMI/IS history were excluded in those studies^41,42^, which the included cases were less than those in real world situation. While some clinical trials demonstrated that IVI did not increase the risk of ATEs compared to the non-IVI groups, patients with cardiovascular diseases, or recent AI or IS were excluded from previous clinical trials^24,41,42^. Malony et al. evaluated the risk of ATEs in DME patients with prior strokes or AMI; the risk of ATEs was not increased in these patients^43^. However, they did not evaluate this risk specifically in patients with an AMI/IS event within 6 months prior to IVI. Thus, our study serves as a reference for future studies involving patients with chronic systemic diseases receiving anti-VEGF agents, particularly those with recent AMI/IS events.

Our study has some limitations. First, the data were obtained from Taiwan’s National Health Insurance Research Database (NHIRD); hence, our findings may not be generalized beyond the study population. Although the results of post hoc analysis performed in the VIEW studies suggest similar levels of safety between white and Asian groups^44^, variations in the features of medical insurance between countries may lead to bias. Taiwan’s health-care system is comprehensive and accessible^45^. Thus, insured patients may not be healthier or financially more secure than noninsured patients. Second, the claims database does not include data regarding fundus fluorescein angiography or optical coherence tomography. To comply with the NHI regulations, health-care providers must upload the medical records of patients for reimbursement applications. However, as mentioned above, NHI approval for reimbursement is based on not only medical coding but also a review of applicants’ medical records. The review process can ensure the validity of the diagnosis code in the NHIRD. Fourth, the detailed reasons for selecting anti-VEGF agents are not recorded in the claims database. Although the guidelines of NHI approval for aflibercept and ranibizumab are the same, there might be some treatment selection bias. Finally, the claims database does not include information regarding smoking history and IS severity, both of which are risk factors for stroke recurrence^46^. Further studies should be conducted to evaluate the correlations of smoking history and IS severity with the risk of major ATEs after the administration of anti-VEGF agents through IVI.

In conclusion, patients receiving IVR exhibit lower risks of ATE than do patients receiving IVA. When devising strategies for IVI with anti-VEGF agents, risk factors such as CAD, RVO, and an AMI/IS event within 6 months prior to treatment must be considered because these factors may increase the risk of ATEs. Thus, caution must be exercised in clinical practice in the treatment of retinal diseases among high-risk patients.

## Data Availability

Data are from the Health and Welfare Science Data Center (HWDC), Ministry of Health and Welfare in Taiwan (http://dep.mohw.gov.tw/DOS/np-2497-113.html). The datasets generated and/or analyzed during the current study are not publicly available due to legal restrictions imposed by the government of Taiwan in relation to the Personal Information Protection Act, but the data are available from the corresponding author on reasonable request.
